# Estimated Exposure to Televised Alcohol Advertisements Among Children and Adolescents

**DOI:** 10.1001/jamanetworkopen.2025.21819

**Published:** 2025-07-17

**Authors:** Yuxiang Tang, Nan Lei, Denghui Hu, Kaipeng Liang, Yang Liu, Tilakavati Karupaiah, Bridget Kelly, Sally Mackay, Boyd Swinburn, Juan Zhang

**Affiliations:** 1 School of Population Medicine and Public Health, Chinese Academy of Medical Sciences and Peking Union Medical College, Beijing, China; 2 National Clinical Research Center for Cardiovascular Diseases, Shenzhen, Fuwai Shenzhen Hospital, Chinese Academy of Medical Sciences, Shenzhen, China; 3 Division of Population Health and Applied Health Sciences, Faculty of Medicine, Memorial University of Newfoundland, Newfoundland and Labrador, Canada; 4 Department of Human Genetics, Leiden University Medical Center, Leiden, the Netherlands; 5 Food Security and Nutrition Impact Lab and Faculty of Health Sciences, National University of Malaysia, Bangi; 6 School of Biosciences, Faculty of Health and Medical Sciences, Taylor’s University, Subang Jaya, Malaysia; 7 Early Start, School of Health and Society, University of Wollongong, Wollongong, Australia; 8 School of Population Health, University of Auckland, Auckland, New Zealand

## Abstract

**Importance:**

Alcohol advertising on television in China has the potential to target children and adolescents with harmful content. Understanding the extent of this advertising is critical for informing and improving current regulatory approaches.

**Objective:**

To measure the exposure of alcohol advertisements on television channels popular among children and adolescents in Beijing, China.

**Design, Setting, and Participants:**

This cross-sectional study of television advertisements used the 4 most popular television channels for viewers aged 3 to 18 years (2 children’s channels and 2 general channels) in Beijing and accessed advertisements recorded from October 19, 2020, to January 17, 2021. Television advertisements were recorded during 4 randomly selected weekdays and 4 randomly selected weekend days (from 6:00 am to 11:59 pm). Data were analyzed from October 1, 2023, to December 31, 2024.

**Exposures:**

Television alcohol advertisements, with food and nonalcoholic beverages (F&B) advertisements classified as not permitted in marketing to children included as comparison.

**Main Outcomes and Measures:**

Primary outcomes included frequency and distribution of alcohol advertisements, rate per channel-hour, and potential exposure during peak viewing times (PVT). Secondary outcomes included comparison with F&B advertisements classified as not permitted based on the World Health Organization Western Pacific Region Office Nutrient Profile Model integrated with the International Network for Food and Obesity/Non-communicable Diseases Research, Monitoring and Action Support (INFORMAS) food classification system and analysis of 6 marketing strategies.

**Results:**

Among 13 864 total advertisements included in the analysis, 5368 were food advertisements. Among the food advertisements, 321 (6.0%; 95% CI, 5.4%-6.7%) were alcohol advertisements and 2001 (37.3%; 95% CI, 36.0%-38.6%) were F&B advertisements classified as not permitted. On general channels, a mean (SD) of 1.1 (1.7) alcohol advertisements per channel-hour were identified, with significantly higher rates during PVT compared with non-PVT (2.0 [2.4] vs 0.7 [0.9] per channel-hour; *P* < .001). The highest rate occurred between 9:00 and 9:59 pm, with a mean (SD) of 3.7 (2.8) advertisements per channel-hour and an estimated mean (SD) of 14 303 014 (11 659 096) impressions among children and adolescents. All 321 alcohol advertisements (100%; 95% CI, 98.9%-100%) and 1997 F&B advertisements classified as not permitted (99.8%; 95% CI, 99.5%-99.9%) used at least 1 marketing strategy, predominantly brand benefit claims, which were used in 307 alcohol advertisements (95.6%; 95% CI, 92.8%-97.4%) and 1915 F&B advertisements classified as not permitted (95.7%; 95% CI, 94.7%-96.5%).

**Conclusions and Relevance:**

In this cross-sectional study of television advertising, alcohol advertisements on general channels exceeded regulatory limits, especially during PVT. These findings suggest that current regulations allow exposure of children and adolescents to alcohol marketing and should be strengthened.

## Key Points

**Question:**

What is the estimated exposure to televised alcohol advertisements among children and adolescents in Beijing?

**Findings:**

In this cross-sectional study of 13 864 advertisements, alcohol advertisements on general channels (mean, 19 per day) exceeded regulatory limits (12 per day). Notably, alcohol advertisement airings peaked between 9:00 and 9:59 pm, with a mean of 3.7 advertisements per channel-hour and an estimated mean of 14.3 million impressions among children and adolescents.

**Meaning:**

These findings suggest that current regulations permitting child and adolescent exposure to televised alcohol advertisements should be strengthened.

## Teaser

This cross-sectional study estimates the rate of alcohol advertisements on television channels popular among children and adolescents in Beijing, China, and compares them with the rates of advertisements for food and nonalcoholic beverages that are not permitted for marketing to children.

## Introduction

Alcohol consumption poses a significant global public health challenge, with particularly concerning trends in China.^1^ Since 1990, alcohol consumption in China has rapidly increased.^2^ This trend is especially alarming among Chinese youths.^3,4,5^ A comprehensive meta-analysis^6^ reported that 23.6% of Chinese adolescents consumed alcohol in the past 30 days and 36.5% in their lifetime, with increases of 4.5 and 8.8 percentage points per decade, respectively. A 2015 survey conducted across 6 Chinese cities^7^ revealed that 18.1% of students reported first alcohol use at or before 7 years of age, while 22.9% initiated alcohol use between 12 and 13 years of age. Given that no level of alcohol consumption is considered safe, and even minimal intake can significantly impair neurological functions in youths, targeted interventions are crucial to prevent early alcohol initiation.^8,9,10,11,12^

Alcohol advertising shapes youths’ attitude toward alcohol and promotes early initiation of use and risky drinking behaviors.^13,14,15^ An Australian study^16^ found that higher levels of prior exposure to television alcohol advertisements were associated with a greater likelihood of alcohol consumption later in adolescence. Each additional hour of daily television viewing increased risk of initiation of alcohol use by 9%, while each additional advertisement viewed correlated with a 1% consumption increase in adolescents.^14^ These findings underscore the pervasive and potentially harmful impact of alcohol advertisements on minors through TV.

In response to these concerns, the World Health Organization (WHO) introduced the SAFER strategy in 2018 to reduce harmful alcohol use. The SAFER framework consists of 5 components: strengthen restrictions on alcohol availability; advance and enforce drunk-driving countermeasures; facilitate access to screening, brief interventions, and treatment; enforce bans or comprehensive restrictions on alcohol advertising, sponsorship, and promotion; and raise prices on alcohol through excise taxes and pricing policies.^17^ The framework identifies enforcing bans or comprehensive restrictions on alcohol advertising as a highly cost-effective “best buy” intervention, particularly important for protecting children and adolescents, and recommends that governments establish robust enforcement mechanisms and effective deterrents to prevent violations of marketing restrictions.^17^

There are several alcohol marketing regulations in China. For example, China implemented the 2015 Advertising Law of the People’s Republic of China, which prohibits alcohol advertisements on mass media platforms targeting minors.^18^ Given that television is the primary marketing medium with a penetration rate of 99.7% in China,^19^ alcohol advertising must also comply with the 2010 Regulation on Broadcasting of Radio and Television Advertisements.^20^ This regulation limits alcohol advertisements to no more than 12 per day on each permitted television channel, with a maximum of 2 between 7:00 and 9:00 pm. Violations of this regulation may incur warnings and fines up to 20 000 CNY (approximately US $2759).^20^ Despite these restrictions, alcohol industries in China spent approximately 28.65 billion CNY (approximately US $3.94 billion) on television advertisements in 2022,^21^ raising concerns about regulatory effectiveness in protecting minors from exposure to alcohol marketing.

This study aimed to estimate alcohol advertisements on television channels popular among children and adolescents in Beijing and compared them with advertisements for food and nonalcoholic beverages (F&B) that are not permitted for marketing to children according to the WHO Western Pacific Region Office Nutrient Profile Model (WPRO NPM) integrated with the International Network for Food and Obesity/Non-communicable Diseases Research, Monitoring and Action Support (INFORMAS) food classification system.^22,23^ These products, considered unhealthy, are regulated differently in China: alcohol advertisements face restrictions, while F&B advertisements classified as not permitted remain unregulated. By comparing these 2 categories, the study sought to highlight gaps in current regulations and provide evidence-based recommendations to better protect children and adolescents from harmful marketing.

## Methods

### Study Design

This cross-sectional observational study was conducted as part of a larger multicountry investigation by the Food Systems Accountability and Transformation Asia (FoodACT Asia) team across 9 Asian countries, with technical support from INFORMAS.^24^ The INFORMAS protocol established standardized data collection to enable cross-country comparability of children’s exposure to unhealthy food marketing on television. We adopted a modified version of the INFORMAS food promotion module’s television protocol,^22^ with adaptation to suit the Chinese context. Details were described in the parent study.^24^ The study was approved by the Institutional Review Board of the Chinese Center for Disease Control and Prevention. This study did not involve human participants and therefore did not require informed consent. This study followed the Strengthening the Reporting of Observational Studies in Epidemiology (STROBE) reporting statement.

### Data Source

All data were obtained from Kuyun EYE Grow (hereafter Kuyun), a third-party media monitoring service in Beijing. The platform monitored real-time viewing data from approximately 80 million smart television set-top boxes across multiple brands as of 2019 in China.^25^ We chose Kuyun due to its broader terminal coverage and automated data collection methods, which minimized human intervention bias. Additionally, Kuyun’s real-time full-sample tracking offered advantages over traditional television audience measurement systems, which rely on smaller panels and may be prone to selection bias.^25^

### Sample Selection and Data Collection

We selected the top 4 popular television channels among children and adolescents aged 3 to 18 years in Beijing based on Kuyun’s viewership data. Note that *Beijing* refers to the location of Kuyun’s sample users, not a geographical boundary, as the channels have nationwide coverage. To validate these data, we conducted an online survey of 193 households and consulted 2 experts in the field. The selected channels consisted of 2 children’s channels and 2 general channels, designated as national children’s channel, local children’s channel, national general channel 1, and national general channel 2. Following the INFORMAS protocol, we randomly selected 4 weekdays and 4 weekend days between October 19, 2020, and January 17, 2021, excluding major festivals, public holidays, and school holidays. Kuyun recorded all television programs on the selected channels from 6:00 am to 11:59 pm on the chosen dates, totaling 576 hours of footage. Timestamps were projected onto the video screen during recording. Kuyun then segmented the footage into hourly clips and identified individual advertisements. For each advertisement, we documented key details, including advertisement and product details, broadcast timing, and programming context, with each advertisement assigned a unique identifier. Additionally, we obtained hourly viewership data for children and adolescents in Beijing for the 4 channels during 3 consecutive months, excluding major festivals, public holidays, and school holidays.

### Data Coding

Food advertisements were coded according to WPRO NPM^23^ integrated with the INFORMAS food classification system.^22^ The rationale for this integration was 2-fold: first, the WPRO NPM was tailored to dietary patterns in the Western Pacific Region (including China), making it contextually appropriate; second, the INFORMAS coding classification complemented it by including categories not covered by the WPRO NPM (eg, infant formula, alcohol, dietary supplements). The integrated food classification system is detailed in eTable 1 in Supplement 1.

The integrated food classification system established threshold standards based on 7 nutrients, including total fat, saturated fatty acids, total sugar, added sugar, nonsugar sweeteners, sodium, and energy. We verified the nutrient content of each product using the nutrition facts panel on the packages or the official manufacturer websites. Due to the widespread absence of total sugar and saturated fatty acid data, which were not required by labeling regulations at the time of study, these indicators were excluded in the classification system in our study. Products were categorized as not permitted (exceeding any nutrient threshold), permitted (meeting all thresholds), not applicable (eg, infant formula), or brand only (for advertisements without a specific product). For multiproduct advertisements (maximum of 3 dominant products coded), if any product failed to meet the criteria, the entire advertisement was classified as not permitted for marketing to children and adolescents. All alcohol advertisements were classified as not permitted.^22,23^

We coded 6 marketing strategies and their associated marketing techniques in alcohol and F&B classified as not permitted. These strategies were adapted from the INFORMAS protocol^22^ and informed by a relevant systematic review^26^ to better reflect the Chinese food marketing context. The strategies included brand benefit claims (highlighting various product advantages and use value), promotional characters (using recognizable characters, events, awards, and targeted imagery to enhance product appeal), premium offers (contests, gifts, or collectible items with purchase), advercation (a combination of advertisement and education, incorporating educational elements about history, nutrition, or product ingredients), claims (various health, nutritional, functional, or natural quality assertions about the product), and marketing partnerships (collaborations with other brands or entities). Adaptations were developed in consultation with the FoodACT Asia team to ensure cultural relevance while maintaining methodological consistency. Detailed marketing techniques for each strategy are presented in eTable 2 in Supplement 1. Following the INFORMAS protocol, we defined peak viewing times (PVT) as the top five 1-hour broadcast periods with the highest child and adolescent viewership.^22^ All other times were classified as non-PVT (NPVT). Based on Kuyun’s viewership data, PVT was identified as 6:00 to 10:59 pm on both weekdays and weekends.

### Reliability

We conducted intercoder reliability (IRR) testing following the established INFORMAS protocol to ensure consistent data coding.^22^ For internal validation, a randomly selected hour of television recording was independently coded by researchers (including Y.T., N.L., and K.L.). IRR was calculated using the formula Agreement/(Agreement + Disagreement) × 100. A minimum threshold of 90% agreement was set. When initial coding fell below this threshold, additional training was provided, followed by repeated testing with different sample hours until the threshold was achieved. Discrepancies were resolved through detailed discussions with reference to the coding protocol and, if necessary, consultation with a third researcher (J.Z.). Once 100% internal consistency was achieved, the data were submitted to the FoodACT Asia team for external validation, with an acceptable IRR range of 80% to 100%.^22^ Our study met these criteria, ensuring robust coding reliability. All inconsistencies were jointly resolved before proceeding with data analysis.

### Statistical Analysis

Data were analyzed from October 1, 2023, to December 31, 2024. Analyses were performed using SPSS, version 26.0 (IBM Corporation). To account for unequal sampling, weekday and weekend data were weighted (1.40 and 3.48, respectively) based on selection probability during the 3-month period (eTable 3 in Supplement 1).^22^ We analyzed alcohol advertisements and F&B advertisements classified as not permitted using frequencies and rates, where rates were calculated as the number of advertisements per channel per hour (ie, per channel-hour). To estimate potential exposure, we multiplied the channel-specific mean number of children and adolescent viewers per hour by the mean number of advertisements classified as not permitted per hour.^27^ The channel-specific mean number of viewers is presented in eTable 4 in Supplement 1. Mann-Whitney testing was used to compare frequencies between weekdays and weekends and between PVT and NPVT. All tests were 2 tailed. Statistical significance was set at *P* < .05.

## Results

### Frequency and Distribution of Advertisements Across Channels

**Figure 1.**
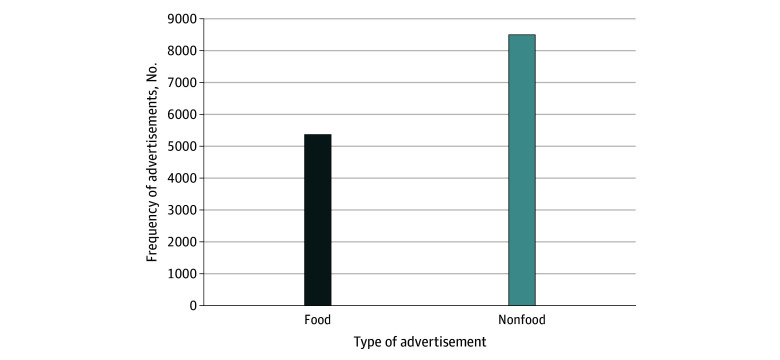
Frequency of Television Advertisements

A total of 13 864 television advertisements were collected, including 5368 food advertisements (38.7%; 95% CI, 37.9%-39.5%) (Figure 1). Among food advertisements, 321 (6.0%; 95% CI, 5.4%-6.7%) promoted alcohol products, all of which were categorized as not permitted for marketing to children and adolescents. The remaining 5047 food advertisements (94.0%; 95% CI, 93.3%-94.6%) were for F&B, with 2001 of these (39.6%; 37.3% [95% CI, 36.0%-38.6%]) classified as not permitted.

Table 1 presents the distribution of advertisements across channel types. All alcohol advertisements were broadcast on general channels, with national general channel 1 airing the highest proportion (263 of 321 [81.9%]). In contrast, most F&B advertisements classified as not permitted (1591 of 2001 [79.5%]) were aired on children’s channels, particularly on the national children’s channel (1280 of 2001 [64.0%]). On general channels, alcohol advertisements constituted 321 of 2099 advertisements (15.3%), while F&B advertisements classified as not permitted accounted for 410 of 2099 (19.5%).

**Table 1.** Frequency and Proportion of Alcohol and F&B Advertisements

Category of advertisement	No. (%) of advertisements [95% CI]^a^						
	Total	National general channel			Children’s channel		
		1	2	Overall	National	Local	Overall
Alcohol (n = 321)							
Not permitted	321 (6.0) [5.4-6.7]	263 (40.9) [37.2-44.7]	58 (4.0) [3.1-5.1]	321 (15.3) [13.8-16.9]	0 [0.0-0.2]^b^	0 [0.0-0.4]^b^	0 [0.0-0.1]^b^
F&B (n = 5047)							
Not permitted	2001 (37.3) [36.0-38.6]	131 (20.4) [17.5-23.7]	279 (19.2) [17.3-21.3]	410 (19.5) [17.9-21.3]	1280 (53.5) [51.5-55.5]	311 (35.5) [32.4-38.7]	1591 (48.7) [47.0-50.4]
Permitted	1408 (26.2) [25.0-27.4]	165 (25.7) [22.5-29.2]	413 (28.4) [26.0-30.7]	578 (27.5) [25.6-29.5]	830 (34.7) [32.8-36.6]	0 [0.0-0.4]^b^	830 (25.4) [23.9-26.9]
Not applicable	1630 (30.4) [29.2-31.6]	80 (12.4) [10.1-15.2]	702 (48.2) [45.6-50.8]	782 (37.3) [35.3-39.4]	284 (11.9) [10.6-13.2]	564 (64.5) [61.3-67.6]	848 (25.9) [24.5-27.5]
Brand only	8 (0.1) [0.0-0.2]	4 (0.6) [0.2-1.6]	4 (0.3) [0.1-0.7]	8 (0.4) [0.2-0.8]	0 [0.0-0.2]^c^	0 [0.0-0.4]^c^	0 [0.0-0.1]^c^
All	5368 (100)	643 (100)	1456 (100)	2099 (100)	2394 (100)	875 (100)	3269 (100)

Abbreviation: F&B, food and nonalcoholic beverages.

^a^ All proportions are presented with 95% CIs calculated using the Wilson score method.

^b^ Zero values in alcohol advertisements in children’s channels reflect the effective implementation of regulatory prohibitions.

^c^ Zero values represent absence of such advertisements in the sampled content, rather than regulatory restrictions.

### Rate of Not Permitted Advertisements Across Channels and Viewing Times

Table 2 displays the rates of advertisements across various television channels and times. The mean (SD) number of alcohol advertisements was 19 (13) per day per channel. On general channels, alcohol advertisements constituted a mean (SD) of 1.1 (1.7) per channel-hour, with significantly higher rates during PVT compared with NPVT (2.0 [2.4] vs 0.7 [0.9] per channel-hour; *P* < .001). Advertisements on general channels for F&B classified as not permitted constituted a mean (SD) of 1.4 (1.6) per channel-hour, with no significant difference between PVT and NPVT. Children’s channels exhibited a significantly higher rate of F&B advertisements classified as not permitted, with a mean (SD) of 6.6 (10.1) per channel-hour. This rate was markedly higher on weekend days (8.1 [11.2] per channel-hour) compared with weekdays (3.0 [5.0] per channel-hour; *P* < .001).

**Table 2.** Mean Rate of Alcohol Advertisements and F&B Advertisements Classified as Not Permitted

Category of channel	Rate, mean (SD), No. of advertisements/channel-hour			*P* value	Rate, mean (SD), No. of advertisements/channel-hour		*P* value
	Total	Weekdays	Weekend days		PVT	NPVT	
**Alcohol advertisements**							
General	1.1 (1.7)	1.2 (1.8)	1.0 (1.5)	.27	2.0 (2.4)	0.7 (0.9)	<.001
National general 1	1.8 (1.9)	2.0 (2.2)	1.7 (1.8)	.41	3.1 (2.8)	1.2 (1.1)	<.001
National general 2	0.4 (0.7)	0.4 (0.7)	0.4 (0.7)	.45	0.8 (1.1)	0.2 (0.4)	.002
**F&B advertisements classified as not permitted**							
General	1.4 (1.6)	1.5 (1.7)	1.4 (1.5)	.60	1.5 (1.5)	1.4 (1.6)	.07
National general 1	1.0 (1.3)	0.8 (1.0)	1.1 (1.4)	.26	1.2 (1.2)	0.9 (1.3)	.001
National general 2	1.8 (1.7)	2.2 (2.0)	1.7 (1.6)	.03	1.8 (1.7)	1.8 (1.8)	.96
Children’s	6.6 (10.1)	3.0 (5.0)	8.1 (11.2)	<.001	4.9 (6.8)	7.3 (11.0)	.80
National	11.1 (12.6)	3.7 (6.6)	14.1 (13.1)	<.001	6.6 (9.0)	12.8 (13.3)	<.001
Local	2.1 (2.0)	2.3 (2.5)	2.1 (1.9)	.99	3.1 (2.5)	1.7 (1.7)	<.001
All	4.0 (7.7)	2.2 (3.8)	4.7 (8.6)	.004	3.2 (5.2)	4.3 (8.4)	.24

Abbreviations: F&B, food and nonalcoholic beverages; NPVT, nonpeak viewing times; PVT, peak viewing times.

### Distribution and Potential Exposure of Not Permitted Advertisements

**Figure 2.**
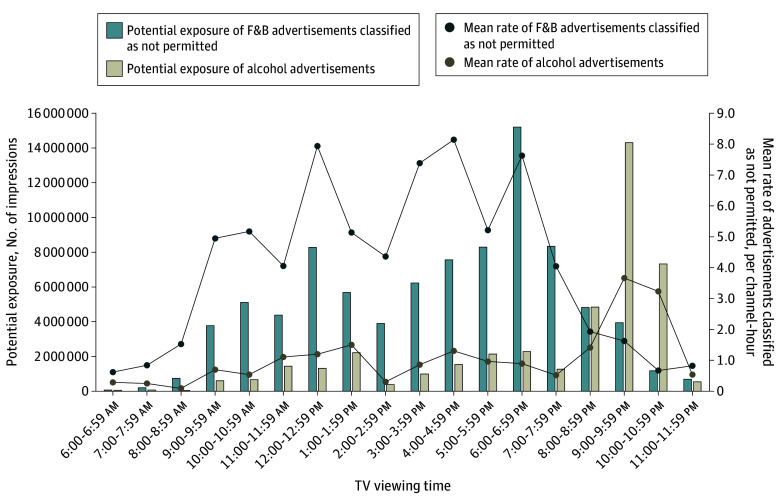
Hourly Exposure to Alcohol Advertisements and Food and Nonalcoholic Beverages (F&B) Advertisements Classified as Not Permitted Potential exposure represents the estimated number of viewer impressions, calculated by multiplying the channel-specific mean number of children and adolescent viewers per hour by the mean number of advertisements per hour. Mean rate represents the mean number of advertisements per channel per hour. Peak viewing time was defined as 6:00 to 10:59 pm, when the rate of alcohol advertisements was significantly higher than during nonpeak viewing time on general channels (*P* < .001).

Figure 2 shows alcohol advertisements peaked between 9:00 and 9:59 pm, with a mean (SD) of 3.7 (2.8) advertisements per channel-hour and an estimated mean (SD) of 14 303 014 (11 659 096) impressions among children and adolescents. Advertisements for F&B classified as not permitted showed 3 distinct peaks throughout the day. These peaks occurred from 12:00 to 12:59 pm with a mean (SD) rate of 7.9 (12.5) advertisements per channel-hour; 4:00 to 4:59 pm with a mean (SD) rate of 8.1 (13.8) advertisements per channel-hour; and 6:00 to 6:59 pm, with a mean (SD) rate of 7.6 (9.2) advertisements per channel-hour. The highest rate for F&B advertisements classified as not permitted occurred between 6:00 and 6:59 pm, with an estimated mean (SD) of 15 203 028 (18 962 079) impressions among youths.

### Marketing Strategies of Food Advertisements Classified as Not Permitted

Table 3 shows that all 321 alcohol advertisements (100%; 95% CI, 98.9%-100%) and 1997 F&B advertisements classified as not permitted (99.8%; 95% CI, 99.5%-99.9%) used at least 1 marketing strategy. Brand benefit claims were the most prevalent strategy in advertisements for both alcohol (95.6%; 95% CI, 92.8%-97.4%) and F&B classified as not permitted (95.7%; 95% CI, 94.7%-96.5%). Within this strategy, emotive claims techniques were most frequently used (alcohol: 58.3% [95% CI, 52.8%-63.6%]; F&B classified as not permitted: 53.1% [95% CI, 50.9%-55.3%]). For alcohol advertisements, advercation was another commonly used marketing strategy (73.2%; 95% CI, 68.1%-77.8%), with historical facts being the most frequently used technique (60.7%; 95% CI, 55.3%-65.9%). For F&B advertisements classified as not permitted, promotional characters were another common marketing strategy (66.9%; 95% CI, 64.8%-68.9%), with the “for kids” technique being the most frequently used (58.2%; 95% CI, 56.0%-60.3%).

**Table 3.** Marketing Strategies Applied in Advertisements Classified as Not Permitted

Marketing strategy	No. (%) of advertisements [95% CI]	
	F&B classified as not permitted (n = 2001)	Alcohol (n = 321)
Brand benefit claims	1915 (95.7) [94.7-96.5]	307 (95.6) [92.8-97.4]
Emotive claims (eg, fun, feelings of popularity)	1062 (53.1) [50.9-55.3]	187 (58.3) [52.8-63.6]
Sensory-based characteristics (eg, taste, texture, appearance, aroma)	1036 (51.8) [49.6-54.0]	135 (42.1) [36.8-47.6]
Suggested users are children or whole family	407 (20.3) [18.6-22.1]	0 [0.0-1.2]
Suggested use	292 (14.6) [13.1-16.2]	1 (0.3) [0.1-1.7]
Puffery	178 (8.9) [7.7-10.2]	89 (27.7) [23.1-32.8]
Convenience	104 (5.2) [4.3-6.3]	0 [0.0-1.2]
Price	64 (3.2) [2.5-4.1]	8 (2.5) [1.3-4.9]
New brand development	25 (1.2) [0.8-1.8]	79 (24.6) [20.2-29.6]
Advercation	847 (42.3) [40.2-44.5]	235 (73.2) [68.1-77.8]
General nutrition	671 (33.5) [31.5-35.6]	0 [0.0-1.2]
Details on product ingredients	668 (33.4) [31.4-35.5]	107 (33.3) [28.4-38.6]
Historical facts	20 (1.0) [0.6-1.5]	195 (60.7) [55.3-65.9]
Promotional characters	1339 (66.9) [64.8-68.9]	107 (33.3) [28.4-38.6]
For kids (eg, image of a child engaging with the product)	1165 (58.2) [56.0-60.3]	1 (0.3) [0.1-1.7]
Cartoon or company-owned character (eg, M&Ms [Mars Inc])	145 (7.2) [6.1-8.4]	0 [0.0-1.2]
Licensed character (eg, Dora the Explorer [Nickelodeon Animation Studio])	90 (4.5) [3.7-5.5]	1 (0.3) [0.1-1.7]
Movie tie-in	67 (3.3) [2.6-4.2]	0 [0.0-1.2]
Celebrity (nonsports)	51 (2.5) [1.9-3.3]	24 (7.5) [5.1-10.9]
Awards	27 (1.3) [0.9-1.9]	105 (32.7) [27.8-38.0]
Amateur sportsperson	4 (0.2) [0.1-0.5]	0 [0.0-1.2]
Nonsports, historical events, or festivals	0 [0.0-0.2]	1 (0.3) [0.1-1.7]
Marketing partnership	241 (12.0) [10.6-13.5]	35 (10.9) [7.9-14.8]
Premium offers	99 (4.9) [4.0-5.9]	0 [0.0-1.2]
Contests	93 (4.6) [3.8-5.6]	0 [0.0-1.2]
Gift or collectable	6 (0.3) [0.1-0.7]	0 (0.0) [0.0-1.2]
Claims^a^	814 (40.7) [38.6-42.9]	NA
Health-related ingredients	467 (23.3) [21.5-25.2]	NA
Nutrient content	356 (17.8) [16.2-19.5]	NA
Nutrient and other function	313 (15.6) [14.1-17.3]	NA
General health	238 (11.9) [10.6-13.4]	NA
Other (eg, organic)	82 (4.1) [3.3-5.1]	NA

Abbreviations: F&B, food and nonalcoholic beverages; NA, not applicable.

^a^ These claims are not applicable to alcohol advertisements due to regulatory prohibitions.

## Discussion

This study provides, to our knowledge, the first comprehensive estimation of children’s and adolescents’ exposure to alcohol advertisements under China’s current regulatory framework, using exposure to advertisements for F&B classified as not permitted as a comparative benchmark. Despite the current regulatory policies, alcohol advertisements on general channels exceeded regulatory limits, especially during PVT.

While the complete absence of alcohol advertisements on children’s channels in China aligns with similar policies in countries such as France^28^ and Australia,^29^ our findings suggest that this measure alone is insufficient. Children and adolescents are likely to be exposed to alcohol advertising on general channels. A major shortcoming of current regulatory policies is their narrow focus on children’s channels, based on the assumption that these are the primary channels accessed by minors. This assumption overlooks the substantial viewership of general channels among children and adolescents, as supported by 2 studies from 2020.^30,31^ Furthermore, the ambiguity in defining “media targeting minors” in Chinese regulations allows the alcohol industry to redirect advertisements to popular general channels that often feature content appealing to children and adolescents. This underscores the need for more comprehensive and clearly defined regulations that reflect actual media exposure patterns among children and adolescents across all channel types.

We observed a mean (SD) of 19 (13) alcohol advertisements per day per channel, exceeding the regulatory limit of 12 advertisements and highlighting gaps in policy enforcement. This issue was more pronounced on national general channel 1, where daily alcohol advertisements ranged from 27 to 48. This widespread noncompliance may indicate insufficient deterrence. Notably, the fine for excessive advertising on general channels (US $2759) is substantially lower than the fine for illegal advertising on children’s channels (US $27 598), further highlighting the need for a more balanced and stringent enforcement.^18,20^ The 3-fold higher frequency of alcohol advertisements during PVT compared with NPVT warrants attention. This pattern aligns with findings from Australia and the US, where alcohol advertising intensifies during periods of high viewership by children and adolescents.^15,32^ Our study reveals a key mismatch between regulatory and actual PVT. While the conventional prime time is between 7:00 and 10:00 pm and the regulation limits alcohol advertisements between 7:00 and 8:59 pm, actual PVT for children and adolescents spans from 6:00 to 10:59 pm. This discrepancy reflects outdated assumptions and potential regulatory blind spots. Similar gaps have been observed in countries such as Australia, where alcohol advertising restrictions fail to align with children’s real viewing habits.^33^ These findings call for evidence-based revisions to current regulations.

Our study revealed the widespread use of persuasive marketing strategies in alcohol advertisements, with every advertisement using at least 1 such strategy. Compared with advertisements for F&B classified as not permitted, which often use overt appeals to kids, alcohol advertisements in China are legally restricted from explicitly targeting minors. However, our findings suggest that the alcohol industry circumvents these restrictions by using more subtle, culturally resonant marketing strategies. Brand benefit claims and advercation were most common, often linking alcohol to positive imagery, cultural heritage, or personal success.^34,35,36^ The frequent use of the historical facts technique under the advercation marketing strategy may create historical and cultural appeals derived from the unique heritage and characteristics of alcohol products or brands.^37^ This approach may be associated with the development of positive attitudes toward alcohol consumption among children and adolescents,^38^ though our study did not directly assess such associations.

This study suggests that despite existing policies, alcohol advertisements remain prominent during times and on channels popularly viewed by children and adolescents, raising concerns about inadequate regulation. This pattern of exposure aligns with international experience, where self-regulation of alcohol marketing has been found to be largely ineffective in protecting children and adolescents from exposure.^39^ The alcohol industry’s profitability and market concentration make self-regulation unlikely to protect high-risk groups, such as children and adolescents.^40^ Evidence from comprehensive bans in Norway and Lithuania have demonstrated public health benefits. For instance, Norway’s 1975 ban correlated with a 7.4% reduction in alcohol sales.^41^ France’s Évin Law provides another regulatory model by prohibiting alcohol advertising on television and restricting permitted media advertisements to objective product characteristics (eg, alcohol content), while prohibiting associations with happiness or social success.^28^ The effectiveness of such content restrictions is supported by existing evidence in the literature,^42^ as a mixed-methods study found these content-restricted advertisements significantly reduced young people’s purchase and consumption intentions, especially those younger than 22 years.

To address the concerning levels of estimated alcohol exposure among children and adolescents identified in our study, we support the WHO recommendations: enforcing bans or comprehensive restrictions on exposure to alcohol advertising can be enacted and enforced by setting upregulatory or coregulatory frameworks with legislative basis.^43^ Given the current situation in China, a phased approach could begin with stricter limits on airtime and volume but ultimately should include broader coverage of all popular viewing periods, tighter content controls, increased penalties for violations, and improved media monitoring systems to track youth exposure.

### Limitations

This study has several limitations. First, our data were limited to households with children and adolescents, which may not fully capture the viewing patterns of children and adolescents in other settings or when they are outside. Second, by focusing solely on spot advertisements during program breaks, we likely underestimated total exposure, as we did not include other forms of television advertising such as sponsorships or product placements. Third, our analysis focused exclusively on traditional television advertising and did not capture alcohol marketing through digital streaming services or social media platforms, which represent increasingly prominent channels for youth media consumption. This may result in underestimation of total alcohol advertising targeting children and adolescents. Fourth, although we recorded when advertisements were broadcast, we cannot confirm whether children and adolescents were actively watching during these periods. Finally, while we identified alcohol advertisements aired during times when young audiences were likely present, we did not measure links with drinking behaviors. Our findings describe advertising patterns and regulatory compliance but do not establish causal relationships. Further research is needed to explore potential associations between advertising exposure and alcohol consumption among youths.

## Conclusions

In this cross-sectional observational study, we observed frequent alcohol advertisements on general channels during PVT, periods when children and adolescents are most likely to be watching television, highlighting inadequacies in the current regulatory framework. These findings underscore the urgent need for more comprehensive and rigorously enforced policies to protect children and adolescents from alcohol marketing exposure. As China continues to grapple with rising rates of alcohol consumption, particularly among children and adolescents, addressing these regulatory gaps should be a public health priority.

## Notes

Supplement 1. **eTable 1.** Integration of WHO WPRO NPM and INFORMAS Food Classification Codes **eTable 2.** Categories of Marketing Strategies and Techniques in Television Advertising **eTable 3.** Weight Calculation for Weekdays and Weekend Days **eTable 4.** Mean Number of Children and Adolescent Viewers per Hour by Channel

Supplement 2.
Data Sharing Statement
