# Supplementary material for: Estimated Exposure to Televised Alcohol Advertisements Among Children and Adolescents
Source: JAMA Netw Open. 2025 Jul 17;8(7):e2521819. doi: 10.1001/jamanetworkopen.2025.21819 (PMC12272293; doi:10.1001/jamanetworkopen.2025.21819)
Supplement: Supplement 1. — eTable 1. Integration of WHO WPRO NPM and INFORMAS Food Classification Codes eTable 2. Categories of Marketing Strategies and Techniques in Television Advertising eTable 3. Weight Calculation for Weekdays and Weekend Days eTable 4. Mean Number of Children and Adolescent Viewers per Hour by Channel [file jamanetwopen-e2521819-s001.pdf]

Supplementary Online Content

Tang Y, Lei N, Hu D, et al. Estimated exposure to televised alcohol advertisements among children and adolescents. *JAMA Netw Open.* 2025;8(7):e2521819. doi:10.1001/jamanetworkopen.2025.21819

eTable 1. Integration of WHO WPRO NPM and INFORMAS Food Classification Codes
eTable 2. Categories of Marketing Strategies and Techniques in Television Advertising
eTable 3. Weight Calculation for Weekdays and Weekend Days
eTable 4. Mean Number of Children and Adolescent Viewers per Hour by Channel

This supplementary material has been provided by the authors to give readers additional information about their work.

© 2025 Tang Y et al. *JAMA Network Open*.

eTable 1. Integration of WHO WPRO NPM and INFORMAS Food Classification Codes

	Food code	Food category	Included in category	Classification codes	Marketing prohibited if exceeds per 100 g						
					Total fat (g)	saturated fat (g)	Total sugars (g)	Added sugars (g)	non-sugar sweetener	Sodium (g)	Energy (kcal)
WHO WPRO NPM	1	Chocolate & sugar confectionery, energy bars, sweet toppings & desserts	Chocolate (including milk, dark and white chocolate), chocolate spread, cereal, granola and muesli bars, hard/chewy candy, table sugars, flour-based confectionaries, spread including peanut butter, chewing gum, caramels, soft jellied candies, marshmallows, honey, puddings, cream desserts	[a] Refer to WPRO criteria	Not permitted						
	2	Cakes, sweet biscuits & pastries, other sweet bakery products, dry mixes for making	Buns with sweet fillings, cookies, breakfast biscuits, donuts	Refer to WPRO criteria	Not permitted						

© 2025 Tang Y et al. *JAMA Network Open*.

	3	Savoury snacks	Popcorn and maize corn, nuts and mixed nuts (including with fruit content), savory biscuits, crackers, pretzels, other snacks made from rice, maize, wheat, dough or potato (i.e. chips, crisps), pork and chicken rind, processed seaweed	Refer to WPRO criteria				0		0.04	
		**Beverages**									
	40	a) Juices	100% fruit and vegetable juices, (including sugar cane juices, juices reconstituted from concentrate, unsweetened fresh coconut juice), smoothies	Refer to WPRO criteria			5		0		
	41	b) Milk drinks	Milks and sweetened milks, reconstituted powdered milk, almond, soya, rice and oat milks, goat milk, condensed milk, milk shakes, sweetened creamer, evaporated milk [inclusive of follow-up powdered milk >36 months]	Refer to WPRO criteria	4		0	0			
	42	c) Energy drinks, tea and coffee	Energy drinks, tea (including instant and premixed tea with caffeine), coffee (including instant and premixed coffee) [Note: No need to reconstitute as the criteria set as 'not permitted'].	Refer to WPRO criteria	Not permitted						

© 2025 Tang Y et al. *JAMA Network Open*.

	43	d) Other beverages	Other sugar-sweetened beverages, including softdrinks/sodas, juice drinks and flavoured waters, reconstituted chocolate or malted powdered drinks, syrups, mineral and/or flavoured waters (including aerated), powdered juices	Refer to WPRO criteria				0	0		
	5	Edible ices	Ice cream, iced lollies and sorbets, frozen fruit juices, frozen yogurt	Refer to WPRO criteria	4		10		0	0.08	
	6	Breakfast cereals	Chocolate breakfast cereals, oatmeals, mueslis, cornflakes	Refer to WPRO criteria	10		15		0	0.64	
	7	Yogurts, sour milk, cream, other similar foods	Yogurt, flavoured sour milk and drinking yogurt, cheese-based and other yogurt substitutes, fermented milk, curds	Refer to WPRO criteria	4		10		0	0.08	
	8	Cheese	Medium-hard and hard cheeses (i.e. cheddar), soft cheese (e.g. ricotta, mozzarella), sliced cheese, cream cheeses, spreadable cheeses, grated or powdered cheese, cottage cheese, processed cheese	Refer to WPRO criteria	20					0.52	

© 2025 Tang Y et al. *JAMA Network Open*.

	9	Ready-made & convenience foods & composite dishes	Pizzas, lasagne, ready-made sandwiches, tinned spaghetti, instant noodles, instant porridge (e.g. congee), baked beans, creamed corn, steamed pork buns, dumplings, burgers in buns, ready meals, filled pastas, soups, French fries, buttered toasted bread	Refer to WPRO criteria	10	4	10			0.4	225
	10	Butter, other fats & oils	Butter, vegetable oils, margarine and spreads, lard, dripping, ghee	Refer to WPRO criteria		20		0	0	0.56	
	11	Bread, bread products and crisp breads	Bread (i.e. white, yellow, whole wheat), rotis, prata, wraps/tortillas, bread with raisins, buns, toast, cheese bread	Refer to WPRO criteria	10		10			0.48	
	12	Fresh or dried noodles, pasta,rice and grains	Dried and fresh noodles, sago, tapioca	Refer to WPRO criteria	10		10			0.48	
	13	Fresh and frozen meat, poultry, fish and similar	Turkey tails, chicken tail, lamb neck, mutton flap, eggs, oily fish (e.g. herring)	Refer to WPRO criteria	20						

© 2025 Tang Y et al. *JAMA Network Open*.

	14	Processed meat, poultry, fish and similar	Sausage, ham, bacon, canned meat (e.g. Spam) and fish, chicken nuggets, fish fingers, fish balls, beef or chicken patty, liver paste	Refer to WPRO criteria	20					0.68	
	15	Fresh and frozen fruit, vegetables & legumes	Potatoes, roots crops, fresh coconut, mushrooms	Refer to WPRO criteria	20					0.68	
	16	Processed fruit, vegetables and legumes	Dried fruit, dried coconut, coconut cream, marmalade, jams, tinned fruits, vegetables and legumes, dried mushrooms, preserved or picked fruits and vegetables, fermented vegetables, mango chutney, Quorn (meat substitute made from fungus)	Refer to WPRO criteria	5		10	0		0.4	
	17	Sauces, dips and dressings	Tomato ketchup, coloured ketchup, mayonnaise, salad dressing, soy sauce, fish sauce, sweet chili sauce, gravies, spaghetti sauce, barbecue sauces, seasonings, reconstituted stocks	Refer to WPRO criteria	10			0		0.4	
	18	Products made from soya	Tofu products, natto, tempeh	Refer to WPRO criteria	12		10	0		0.4	
INFORMAS	19	Alcohol		Code as 0 = Not permitted	Not permitted						

© 2025 Tang Y et al. *JAMA Network Open*.

food classification system	20	Baby foods (Exclude milk formulae)		Code as 2 = Not applicable	Not applicable
	21	Baby formulae	<12 months.<br>[Note: This includes Step 2 formula - 6 - 18 months].	Code as 2 = Not applicable	Not applicable
	22	Follow up formulae	12 - 36 months.<br>[Note: For follow-up formulate >36 months [e.g. Step 4 (4-6 years); Step 5 (6 years and above)], this will be coded under "WPRO code 41"].	Code as 2 = Not applicable	Not applicable
	23	Fast-food restaurant (NO foods or drinks advertised)		Code as 4 = Brand only	Company brand only without food products
	24	Local restaurant (No foods or drinks advertised)		Code as 4 = Brand only	Company brand only without food products

© 2025 Tang Y et al. *JAMA Network Open*.

	25	Supermarkets (NO foods or drinks advertised)		Code as 4 = Brand only	Company brand only without food products
	26	Vitamin/ mineral or other dietary supplements	Including nourishing fluids.	Code as 2 = Not applicable	Not applicable
	27	Manufacturer (No foods or drinks advertised)		Code as 4 = Brand only	Company brand only without food products
	0	Food or drink delivery (No foods or drinks advertised)	E.g., UberEat without food product, just brand.	Code as 4 = Brand only	Company brand only without food products

Abbreviations: WHO WPRO, World Health Organization Western Pacific Region Office nutrient profile model (WHO WPRO NPM); INFORMAS, International Network for Food and Obesity/NCD Research, Monitoring and Action Support. [a] Refer to WPRO criteria: Code as 0 = Not permitted, Code as 1 = permitted, Code as 2 = Not applicable, Code as 4 = Brand only

© 2025 Tang Y et al. *JAMA Network Open*.

eTable 2. Categories of Marketing Strategies and Techniques in Television Advertising

Marketing strategies	Marketing techniques
Brand benefit claims	Emotive claims (fun, feelings, popularity)
	Sensory based characteristics (taste, texture, appearance, aroma)
	Suggested users are children or whole family
	Suggested use (e.g., great for lunchboxes)
	Puffery (e.g., claiming to be advantageous over other)
	Convenience
	Price
	New brand development
Advercation	General nutrition (e.g., Fermentation aids digestion)
	Details on product ingredients (e.g., cocoa production for chocolate)
	Historical facts (e.g., 4000 years of cultivation history)
Promotional characters	"For kids" (e.g., image of a child engaging with the product/ consuming the product, "great for school lunches", "for school lunchboxes")
	Cartoon/Company owned character (e.g., M&Ms)
	Licenced character (e.g., Dora the explorer)
	Movie tie-in (e.g., Shrek)
	Celebrity (non-sports)
	Awards (e.g., Best Food Award 2014, award winning, number one best-selling)
	Amateur sportsperson
	Non-sports/historical events/festivals
Marketing partnership	
Premium offers	Contests
	Gift or collectable
Claims	Health related ingredients claims
	Nutrient content claims (e.g., low fat)
	Nutrient & other function claim
	General health claims (e.g., healthy diet)
	Other claims (e.g., organic)

© 2025 Tang Y et al. *JAMA Network Open*.

eTable 3. Weight Calculation for Weekdays and Weekend Days

Day	Number of days	Days of selection	Chance of selection [a]	Weight [b]
weekday	62	4	0.71	1.40
weekend day	25	4	0.29	3.48
total days	87	8	1.00	/

[a]Chance of selection = number of weekdays or weekend days / total days; [b]Weight=1/Chance of selection (weekdays or weekend days)

© 2025 Tang Y et al. *JAMA Network Open*.

eTable 4. Mean Number of Children and Adolescent Viewers per Hour by Channel

timeslot	National children's channel	Local children's channel	National general channel 1	National general channel 2
06:00:00--06:59:59	73542	18400	99137	247617
07:00:00--07:59:59	324660	72615	270140	270799
08:00:00--08:59:59	691621	165107	520531	569278
09:00:00--09:59:59	1055128	266706	826898	904024
10:00:00--10:59:59	1129434	322434	1345979	1149712
11:00:00--11:59:59	1300038	425417	1232602	1363886
12:00:00--12:59:59	1439759	528190	914472	1289036
13:00:00--13:59:59	1044890	421139	1633643	1321080
14:00:00--14:59:59	797025	293818	1142856	1339739
15:00:00--15:59:59	791887	277118	1126620	1180460
16:00:00--16:59:59	998059	361697	1147988	1206018
17:00:00--17:59:59	1485313	443856	1685569	2749546
18:00:00--18:59:59	2137099	719365	2581306	2536021
19:00:00--19:59:59	2502855	853582	2664151	2226243
20:00:00--20:59:59	2378670	761921	3706258	3156942
21:00:00--21:59:59	1441451	456858	3812653	3997013
22:00:00--22:59:59	2238124	244796	2290686	2241336
23:00:00--23:59:59	1250891	87505	1035766	975504

© 2025 Tang Y et al. *JAMA Network Open*.
