# Supplementary material for: Estimated Exposure to Televised Alcohol Advertisements Among Children and Adolescents
Source: JAMA Netw Open. 2025 Jul 17;8(7):e2521819. doi: 10.1001/jamanetworkopen.2025.21819 (PMC12272293; doi:10.1001/jamanetworkopen.2025.21819)
Supplement: Supplement 2. — Data Sharing Statement [file jamanetwopen-e2521819-s002.pdf]

Data Sharing Statement

Tang. Estimated Exposure to Televised Alcohol Advertisements Among Children and Adolescents in China. *JAMA Netw Open*. Published July 17, 2025.
doi:10.1001/jamanetworkopen.2025.21819

Data
Data available: Yes
Data types: Data (not involving human participants)
How to access data: The data that support the findings of this study are available from the corresponding author upon reasonable request. Requests may be submitted to zhangjuan@sph.pumc.edu.cn.
When available: With publication

Supporting Documents
Document types: None

Additional Information
Who can access the data: researchers for non-commercial use
Types of analyses: aggregated data rather than raw data
Mechanisms of data availability: This will be decided on a case by case basis.
